# Predictors of treatment outcomes following treat-and-extend regimen with aflibercept for branch retinal vein occlusion: post-hoc analysis of the PLATON trial

**DOI:** 10.1038/s41598-023-38955-4

**Published:** 2023-07-20

**Authors:** Wonyung Son, Woo Jin Jeong, Jung Min Park, Jung-Yeul Kim, Yong-Sok Ji, Min Sagong

**Affiliations:** 1 Department of Ophthalmology, Yeungnam University College of Medicine, #170 Hyunchungro, Nam-gu, Daegu, 42415 Republic of Korea; 2 Department of Ophthalmology, Soonchunhyang University Gumi Hospital, Gumi, Republic of Korea; 3 Department of Ophthalmology, Dong-A University College of Medicine, Busan, Republic of Korea; 4 Department of Ophthalmology, Maryknoll General Hospital, Busan, Republic of Korea; 5 Department of Ophthalmology, Chungnam National University College of Medicine, Daejeon, Republic of Korea; 6 1.0 Eye Clinic, Daejeon, Republic of Korea; 7 Department of Ophthalmology, Chonnam National University Medical School and Hospital, Gwangju, Republic of Korea; 8 Yeungnam Eye Center, Yeungnam University Hospital, Daegu, Republic of Korea

**Keywords:** Retinal diseases, Predictive markers

## Abstract

We investigated predictors of visual outcomes and injection interval in macular edema (ME) secondary to branch retinal vein occlusion (BRVO) treated with a treat-and-extend (TAE) regimen. All 48 patients in a multicenter study were followed for 52 weeks and received three monthly intravitreal aflibercept injections before the TAE regimen, with treatment intervals adjusted by 4 weeks, up to a maximum of 16 weeks. Various laboratory biomarkers and optical coherence tomography parameters were evaluated. Patients were classified into the extension failure group if they had ≥ 1 treatment interval decreased due to an increase in the central macular thickness compared to the previous visit and 18 patients were assigned to this group. In multivariate logistic analyses, presence of microaneurysms and prominent middle limiting membrane (p-MLM) sign, increased initial external limiting membrane (ELM) disruption, and higher total cholesterol were correlated with inhibiting a sustained extension in the injection interval (*P* = 0.015, *P* = 0.032, *P* = 0.037, *P* = 0.009, respectively). Therefore, in the patients with ME secondary to BRVO with these risk factors, early consideration of frequent injection may improve treatment outcome.

## Introduction

Branch retinal vein occlusion (BRVO) is one of the most common retinal vascular diseases that may lead to macular edema (ME) and retinal ischemia^1^. ME associated with BRVO is an important cause of visual impairment. The pathogenesis of ME in BRVO involves the disruption of the blood-retina barrier (BRB) due to retinal ischemia, leading to the release of inflammatory factors such as vascular endothelial growth factor (VEGF), interleukin (IL)-6, IL-8, IL-12, and IL-13. These inflammatory mediators contribute to increased vascular permeability, causing a fluid shift from the vessel components to the retinal cellular components, ultimately leading to the development of secondary ME^2,3^. Many large-scale clinical trials have demonstrated that anti-VEGF therapy has been shown to be an effective and safe treatment for ME due to BRVO^4–6^, however, an optimal protocol for anti-VEGF therapy has not been established.

Several randomized clinical trials (RCTs)^6,7^ such as Branch Retinal Vein Occlusion (BRAVO) trial^8^ used protocol of an initial 6 monthly anti-VEGF injections switching to a pro re nata (PRN; as-needed) regimen with fixed follow-up of monthly intervals. The improvement of best-corrected visual acuity (BCVA) measured using the Early Treatment Diabetic Retinopathy Study (ETDRS) letter score was superior in the BRAVO study (19.6 letters) to other real-world study^9^ with PRN strategy (11.2 letters) at 12 months. However, these RCT results are difficult to replicate in real-world practice because fewer loading injections were performed, and clinicians often accept a minimal fluid to alleviate the substantial patient burden. Therefore, an individualized administration regimen is desirable to minimize the burden of frequent treatment.

A treat-and-extend (TAE) regimen is an effective method that aims to individualize the treatment intervals depending on the presence of ME^10^. The TAE regimen has mainly been used for the administration of anti-VEGF therapy to treat age-related macular degeneration^11^ and several studies have reported the efficacy of a TAE regimen for ME due to BRVO^12,13^. Also, we previously reported the primary outcomes of BCVA improvement and decreased central macular thickness (CMT) in Prospective triaL of a TAE regimen with AflibercepT for macular edema secondary to branch retinal vein OcclusioN (PLATON) trial^14^. However, there has been no investigation for parameters affecting treatment interval in BRVO-ME with the TAE regimen.

In this study, to evaluate predictors of the visual outcome and the injection interval in BRVO-ME treated with the TAE regimen, we conducted comprehensive analysis of spectral domain optical coherence tomography (SD-OCT) parameters and clinical laboratory biomarkers.

## Results

### Baseline demographics

Fifty eyes from 50 patients were included in the study between October 2017 and February 2019. In total, 48 patients completed the study, and two patients withdrew consent from the study before the 52-week endpoint. Table 1 shows the patients’ baseline characteristics and clinical data. The mean age, baseline BCVA, and baseline CMT of the patients were 63.3 ± 11.3 years, 52.5 ± 13.6 letters, and 577.4 ± 236.4 μm, respectively.

**Table 1:** Patient characteristics.

Characteristics	Overall	Failure to maintain a sustained extension of interval		Failure to maintain the maximum interval at 52 weeks	
		E–F group	E–S group	M–F group	M–S group
Eyes (%)	48 (100)	18 (37.5)	30 (62.5)	14 (29.2)	34 (70.8)
Age (year)	63.3 ± 11.3	61.5 ± 8.2	64.5 ± 12.9	60.7 ± 9.0	64.4 ± 12.1
Sex, male/female	21/27	8/10	13/17	6/8	15/19
Presence of HTN, n (%)	17 (35.4)	5 (27.8)	12 (40.0)	4 (28.5)	13 (38.2)
Presence of DM, n (%)	10 (20.8)	2 (11.1)	8 (26.6)	2 (14.2)	8 (23.5)
HbA1c (%)	5.7 ± 0.6	5.54 ± 0.4	5.8 ± 0.9	5.6 ± 0.3	5.7 ± 0.6
Total cholesterol (mmol/l)	192.1 ± 36.5	209.0 ± 35.5	180.3 ± 33.9	197.5 ± 30.3	189.1 ± 39.3
LDL cholesterol (mg/dL)	119.7 ± 31.1	131.7 ± 32.4	111.8 ± 25.4	122.3 ± 27.6	117.1 ± 32.1
Triglyceride (mg/dL)	167.6 ± 117.3	190.6 ± 158.2	157.5 ± 80.2	214.9 ± 170.9	149.2 ± 83.0
HDL cholesterol (mg/dL)	51.8 ± 12.0	52.1 ± 11.2	50.9 ± 11.9	51.8 ± 10.9	51.6 ± 12.7
Baseline BCVA (ETDRS letters)	52.5 ± 13.6	55.2 ± 11.0	50.9 ± 14.9	52.7 ± 15.9	51.0 ± 15.5
Baseline CMT (μm)	577.4 ± 236.4	659.9 ± 307.8	527.9 ± 168.1	698.3 ± 323.9	527.5 ± 171.6
No. of total IAI (n)	6.7 ± 1.2	7.7 ± 1.6	6.1 ± 0.3	8.1 ± 1.5	6.1 ± 0.3
Final IAI interval (week)	14.4 ± 2.8	11.8 ± 3.2	16.0 ± 0.0	8.8 ± 2.7	16.0 ± 0.0
Final BCVA (ETDRS letters)	76.1 ± 10.7	77.8 ± 7.7	75.1 ± 12.2	74.8 ± 11.8	76.6 ± 10.4

*E–F* extension failure, *E-S* extension success, *M-F* maintenance failure, *M-S* maintenance success, *HTN* hypertension, *DM* diabetes mellitus, *HbA1c* glycosylated hemoglobin, *LDL* low-density lipoprotein, *HDL* high-density lipoprotein, *BCVA* best-corrected visual acuity, *ETDRS* early treatment diabetic retinopathy study, *CMT* central macular thickness, *IAI* intravitreal aflibercept injection.

The proportion of patients in the extension failure (E–F) group was 37.5%, and the average period at which patients first failed to extend the interval was 26.9 ± 11.5 weeks. No significant differences were found in clinical laboratory biomarkers between the E–F and extension success (E-S) groups. The total number of injections in the E–F group was higher than in the E-S group (7.7 ± 1.6 vs. 6.1 ± 0.3, *P* = 0.006). The injection interval at the final visit was shorter for the E–F group than that for the E-S group (11.8 ± 3.2 vs. 16.0 ± 0.0, *P* < 0.001). There was no difference of the final BCVA between the E–F and E–S groups (77.8 ± 7.7 vs. 75.1 ± 12.2, *P* = 0.342).

Fourteen of 48 patients (29.2%) failed to maintain the interval of 16-week at 52-week. No significant differences were found between the maintenance failure (M-F) and maintenance success (M-S) groups in clinical laboratory biomarkers. The total number of injections in the M-F group was higher than in the M-S group (8.1 ± 1.5 vs. 6.1 ± 0.3, *P* < 0.001). The injection interval at the final visit was 8.8 ± 2.7 weeks in the M-F group and 16.0 ± 0.0 in the M-S group (*P* < 0.001). The final BCVA did not differ between the M-F and M-S groups (74.8 ± 11.8 vs. 76.6 ± 10.4, *P* = 0.632).

### Association between SD-OCT parameters and baseline BCVA

In the univariate analysis, baseline BCVA was associated with baseline SD-OCT parameters such as CMT, amount of hyperreflective foci (HF), size of the cyst, extent of disorganizations of the retinal inner layers (DRIL), disruption of external limiting membrane (ELM), ellipsoid zone (EZ), and interdigitation zone (IZ) (Table 2). In stepwise multivariate regression analysis, an increase in baseline CMT and DRIL extent was significantly related to low baseline BCVA (*P* < 0.001 and *P* = 0.025, respectively) (Table 2).

**Table 2:** Linear regression analysis for association between baseline SD-OCT parameters and baseline BCVA.

Baseline SD-OCT parameters	Univariable		Multivariable	
	Point estimate (95% CI)	P-value*	Point estimate (95% CI)	P-value*
CMT (μm)	− 0.029 (− 0.044 to − 0.105)	< 0.001	− 0.023 (− 0.038 to − 0.007)	< 0.001
Amount of HF (5 increase)	− 3.052 (− 5.454 to − 0.650)	0.014		
Cyst size (50 μm increase)	− 0.949 (− 1.844 to − 0.053)	0.038		
Presence of microaneurysms	3.548 (− 4.852 to 11.948)	0.399		
Presence of p-MLM sign	− 5.333 (− 15.222 to − 4.555)	0.283		
Presence of microaneurysms	3.548 (− 4.852 to 11.948)	0.399		
Presence of epiretinal membrane	− 1.560 (− 16.220 to 13.101)	0.831		
Presence of subretinal fluid	− 2.598 (− 10.835 to 5.638)	0.528		
DRIL per 100 μm	− 3.066 (− 4.853 to − 1.279)	0.001	− 2.076 (− 3.874 to − 0.277)	0.025
ELM disruption per 100 μm	− 1.758 (− 3.223 to − 0.294)	0.020		
EZ disruption per 100 μm	− 1.647 (− 2.771 to − 0.524)	0.005		
IZ disruption per 100 μm	− 1.348 (− 2.612 to − 0.083)	0.037		

*SD-OCT* spectral domain-optical coherence tomography, *BCVA* best-corrected visual acuity, *CMT* central macular thickness, *HF* hyperreflective foci, *p-MLM* prominent middle limiting membrane, *DRIL* disorganization of retinal inner layers, *ELM* external limiting membrane, *EZ* ellipsoid zone, *IZ* interdigitation zone.

### Clinical biomarkers and SD-OCT parameters associated with 52-week change in BCVA

**Figure 1:**
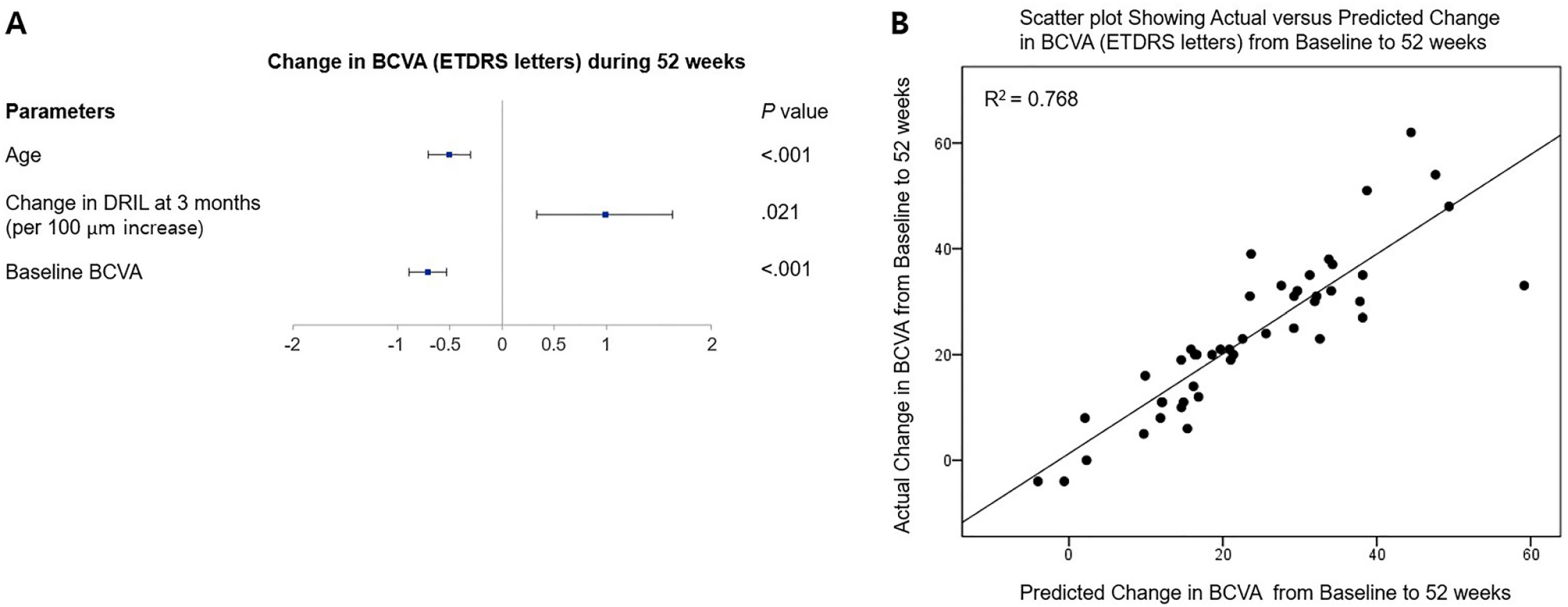
Multivariate analyses of biomarkers and best-corrected visual acuity (BCVA) change during 52 weeks. (**A**) Forest plot showing the result from multivariate stepwise linear regression analyzed statistically significant parameters in univariate linear regression versus BCVA change adjusted for baseline BCVA. Lower baseline BCVA, younger age, and improved DRIL in 3 months were significantly associated with BCVA change *(P* < 0.05, respectively, statistically significant). (**B**) Scatterplot showing actual versus predicted change in BCVA from the baseline to 52-week. *ETDRS* early treatment of diabetic retinopathy study, *DRIL* disorganization of retinal inner layers.

To analyze the multifaceted factors related to BCVA improvement, we included variables among clinical biomarkers, in addition to changes in SD-OCT parameters (Table 3). In univariate analyses, age, HbA1c, baseline BCVA, CMT, amount of HF, the presence of prominent middle limiting membrane (p-MLM), and 3-month change in the extent of DRIL and ELM disruption were associated with BCVA change. However, in stepwise multivariate regression analyses, lower baseline BCVA, younger age, and recovery of DRIL over 3 months were associated with BCVA improvement after aflibercept treatment (*P* < 0.001, *P* < 0.001, and *P* = 0.021, respectively; Fig. 1A). A prediction model combining age and 3-month changes in BCVA and DRIL accounted for 76.8% of the variability in the 52-week BCVA change (Fig. 1B).

**Table 3:** Linear regression analysis for correlation with the 52-week change in BCVA.

Predictors	Univariable		Multivariable	
	Point estimate (95% CI)	P-value	Point estimate (95% CI)	P-value
Age	− 0.458 (− 0.815 to − 0.101)	0.013	− 0.503 (− 0.722 to − 0.283)	< 0.001
Sex, male/female	− 3.496 (− 12.235 to 5.243)	0.424		
Presence of HTN, n (%)	− 8.067 (− 16.769 to 0.636)	0.068		
Presence of DM, n (%)	− 8.017 (− 18.313 to 2.279)	0.124		
HbA1c (%)	− 7.809 (− 14.800 to − 0.818)	0.029		
Total cholesterol (mmol/l)	− 0.008 (− 0.127 to 0.111)	0.894		
LDL cholesterol (mg/dL)	0.016 (− 0.126 to 0.159)	0.816		
Triglyceride (mg/dL)	0.014 (− 0.023 to 0.051)	0.443		
HDL cholesterol (mg/dL)	− 0.340 (− 0.695 to 0.016)	0.060		
Baseline BCVA (ETDRS letters)	− 0.744 (− 0.972 to − 0.516)	< 0.001	− 0.713 (− 0.902 to − 0.524)	< 0.001
Baseline SD-OCT parameter				
CMT (μm)	0.028 (0.012 to 0.044)	0.001		
Amount of HF (5 increase)	2.735 (0.199 to 5.272)	0.035		
Cyst size (50 μm increase)	0.803 (− 0.160 to 1.766)	0.100		
Presence of microaneurysms	− 1.198 (− 10.133 to 7.736)	0.788		
Presence of p-MLM sign	10.256 (0.0145 to 20.366)	0.047		
Presence of epiretinal membrane	− 4.024 (− 19.463 to 11.416)	0.602		
Presence of subretinal fluid	3.413 (− 5.262 to 12.087)	0.432		
DRIL per 100 μm	1.756 (− 0.303 to 3.816)	0.093		
ELM disruption per 100 μm	1.267 (− 0.334 to 2.867)	0.118		
EZ disruption per 100 μm	0.460 (− 0.831 to 1.751)	0.477		
IZ disruption per 100 μm	0.423 (− 0.975 to 1.820)	0.545		
Change of SD-OCT parameters for 3 months				
DRIL per 100 μm	2.339 (1.005 to 3.673)	0.001	1.001 (0.325 to 1.677)	0.021
ELM disruption per 100 μm	1.439 (0.237 to 2.640)	0.020		
EZ disruption per 100 μm	0.302 (− 0.702 to 1.305)	0.547		
IZ disruption per 100 μm	0.641 (− 0.590 to 1.873)	0.299		

*BCVA* best-corrected visual acuity, *HTN* hypertension, *DM* diabetes mellitus, *HbA1c* glycosylated hemoglobin, *LDL* low-density lipoprotein, *HDL* high-density lipoprotein, *BCVA* best-corrected visual acuity, *ETDRS* early treatment diabetic retinopathy study, *SD-OCT* spectral domain-optical coherence tomography, *CMT* central macular thickness, *HF* hyperreflective foci, *p-MLM* prominent middle limiting membrane, *DRIL* disorganization of retinal inner layers, *ELM* external limiting membrane, *EZ* ellipsoid zone, *IZ* interdigitation zone.

### Predictors of frequent injection requirement in the TAE management

Stepwise multivariate logistic regression demonstrated that higher total cholesterol, increased extent of ELM disruption at baseline, presence of microaneurysms, and p-MLM sign were significantly associated with failure to continuously extend the treatment interval (Table 4). Also, the increased extent of ELM disruption at baseline and the presence of microaneurysms were risk factors of failure in maintaining the interval of 16-week at 52-week (Table 5).

**Table 4:** Logistic regression analysis of predictors for failure in sustained extension of the treatment interval in macular edema secondary to BRVO using treat-and-extend regimen.

Predictors	Univariable			Multivariable	
	Odds ratio (95% CI)	P-value		Odds ratio (95% CI)	P-value
Age (year)	0.981 (0.930–1.034)	0.466			
Sex, male/female	0.938 (0.284–3.093)	0.916			
Presence of HTN, n (%)	0.513 (0.143–1.834)	0.304			
Presence of DM, n (%)	0.313 (0.058–1.682)	0.176			
HbA1c (%)	0.346 (0.086–1.385)	0.133			
Total cholesterol (mmol/l)	1.026 (1.005–1.046)	0.014		1.036 (1.009–1.064)	0.009
LDL cholesterol (mg/dL)	1.025 (1.003–1.048)	0.028			
Triglyceride (mg/dL)	1.002 (0.997–1.008)	0.381			
HDL cholesterol (mg/dL)	1.009 (0.960–1.061)	0.731			
Baseline BCVA (ETDRS letters)	1.029 (0.981–1.079)	0.239			
Baseline SD-OCT parameters					
CMT (μm)	1.002 (1.000–1.005)	0.083			
Amount of HF (5 increase)	1.043 (0.597–1.823)	0.882			
Cyst size per (50 μm increase)	0.944 (0.822–1.084)	0.412			
Presence of microaneurysms	7.333 (1.935to 27.794)	0.003		11.058 (1.608–76.061)	0.015
Presence of p-MLM sign	10.400 (1.876–57.648)	0.007		14.865 (1.266–174.538)	0.032
Presence of epiretinal membrane	1.625 (0.208–12.705)	0.644			
Presence of subretinal fluid	1.667 (0.505–5.498)	0.402			
DRIL per 100 μm	1.013 (0.759–1.353)	0.929			
ELM disruption length per 100 μm	1.366 (1.015–1.769)	0.018		1.459 (1.023–2.080)	0.037
EZ disruption length per 100 μm	1.006 (0.844–1.200)	0.944			
IZ disruption length per 100 μm	1.037 (0.856–1.256)	0.712			
Change of SD-OCT parameters for 3 months					
DRIL per 100 μm	0.840 (0.671–1.052)	0.129			
ELM disruption per 100 μm	0.982 (0.825–1.170)	0.842			
EZ disruption per 100 μm	0.929 (0.805–1.073)	0.317			
IZ disruption per 100 μm	1.162 (0.963–1.403)	0.118			

*BRVO* branch retinal vein occlusion, *HTN* hypertension, *DM* diabetes mellitus, *HbA1c* glycosylated hemoglobin, *LDL* low-density lipoprotein, *HDL* high-density lipoprotein, *BCVA* best-corrected visual acuity, *ETDRS* early treatment diabetic retinopathy study, *SD-OCT* spectral domain-optical coherence tomography, *CMT* central macular thickness, *HF* hyperreflective foci, *p-MLM* prominent middle limiting membrane, *DRIL* disorganization of retinal inner layers, *ELM* external limiting membrane, *EZ* ellipsoid zone, *IZ* interdigitation zone.

**Table 5:** Logistic regression analysis of predictors for failure to maintain the maximum interval at 52 weeks in macular edema secondary to BRVO using treat-and-extend regimen.

Predictors	Univariable		Multivariable	
	Odds ratio (95% CI)	P-value	Odds ratio (95% CI)	P-value
Age (year)	0.970 (0.916–1.027)	0.298		
Sex, male/female	1.053 (0.300–3.698)	0.936		
Presence of HTN, n (%)	0.646 (0.167–2.493)	0.526		
Presence of DM, n (%)	0.542 (0.100–2.947)	0.478		
HbA1c (%)	0.635 (0.187–2.161)	0.467		
Total cholesterol (mmol/l)	1.006 (0.989–1.024)	0.472		
LDL cholesterol (mg/dL)	1.006 (0.985–1.026)	0.594		
Triglyceride (mg/dL)	1.005 (0.999–1.011)	0.125		
HDL cholesterol (mg/dL)	1.001 (0.950–1.054)	0.969		
Baseline BCVA (ETDRS letters)	1.003 (0.963–1.045)	0.876		
Baseline SD-OCT parameters				
CMT (μm)	1.003 (1.000–1.006)	0.035		
Amount of HF (5 increase)	1.347 (0.907–2.000)	0.140		
Cyst size per (50 μm increase)	0.958 (0.827–1.108)	0.561		
Presence of microaneurysms	4.600 (1.207–17.524)	0.025	4.485 (1.074–18.723)	0.040
Presence of p-MLM sign	3.000 (0.703–12.803)	0.138		
Presence of epiretinal membrane	0.744 (0.071–7.843)	0.805		
Presence of subretinal fluid	1.714 (0.482–6.093)	0.405		
DRIL per 100 μm	1.095 (0.804–1.491)	0.565		
ELM disruption length per 100 μm	1.348 (1.040–1.748)	0.024	1.343 (1.021–1.767)	0.035
EZ disruption length per 100 μm	1.081 (0.897–1.302)	0.414		
IZ disruption length per 100 μm	0.919 (0.750–1.127)	0.417		
Change of SD-OCT parameters for 3 months				
DRIL per 100 μm	0.889 (0.705–1.121)	0.318		
ELM disruption per 100 μm	1.058 (0.885–1.266	0.537		
EZ disruption per 100 μm	0.962 (0.827–1.119)	0.617		
IZ disruption per 100 μm	0.986 (0.823–1.181)	0.880		

*BRVO* branch retinal vein occlusion, *HTN* hypertension, *DM* diabetes mellitus, *HbA1c* glycosylated hemoglobin, *LDL* low-density lipoprotein, *HDL* high-density lipoprotein, *BCVA* best-corrected visual acuity, *ETDRS* early treatment diabetic retinopathy study, *SD-OCT* spectral domain-optical coherence tomography, *CMT* central macular thickness, *HF* hyperreflective foci, *p-MLM* prominent middle limiting membrane, *DRIL* disorganization of retinal inner layers, *ELM* external limiting membrane, *EZ* ellipsoid zone, *IZ* interdigitation zone.

## Discussion

The development of SD-OCT allows clinicians to quantitatively analyze disturbed foveal photoreceptor integrity in retinal diseases. For this reason, in prior studies, OCT imaging indicators such as DRIL, ELM, and EZ disruption have been suggested as potential biomarkers in RVO after anti-VEGF treatment^15–17^. However, all of these studies performed the PRN regimen and most studies analyzed retrospective data. In our previous study, PLATON^14^, we prospectively applied the TAE regimen to BRVO-ME patients using an aflibercept single agent. We performed a post-hoc analysis of this well-designed study and analyzed the predictors of the visual outcome and change in the injection interval. Also, we included clinically accessible blood-based biomarkers for comprehensive analysis. This study provides useful information for clinicians considering proactive treatment such as TAE regimen for BRVO-ME.

The present study showed that in BRVO-ME, patients with younger ages and low baseline BCVA could reach better visual improvement. Among the SD-OCT parameters, only DRIL improvement in the initial 3 months was correlated with 52-week visual improvement independent of major confounders of visual outcomes such as age and baseline BCVA. Presence of sign of microaneurysm and p-MLM and ELM disruption were SD-OCT parameters of frequent injections as they were limiting factors for sustained extension of the injection interval in BRVO-ME treated with the TAE strategy. Moreover, high serum total cholesterol was the only impediment in laboratory biomarkers to extend the treatment interval continuously.

In other RVO studies,^15,18,19^ age and baseline BCVA have also been identified as important predictive factors for visual potential. Sirakaya et al.^20^ showed that in the short-term response during aflibercept 3-monthly injections in patients with BRVO grouped by age, BCVA improvement and age were negatively correlated. In younger patients, photoreceptors may be less vulnerable to a vascular impairment or mechanical stress due to edema and have enhanced functional and anatomical recovery ability. Paradoxically, patients with a good initial BCVA, expected to have less anatomical or functional damage, have a greater limitation in visual gain after injection treatment. This could be demonstrated by the ceiling effect which has been explained previously^19^. Yiu et al.^15^ also demonstrated that better baseline BCVA was associated with limited visual recovery. This result seems to be because patients with better baseline BCVA had less room for visual gain after treatment, and on the contrary, patients with worse baseline BCVA showed dramatic improvement in BCVA after injection.

In a previous large-scale multicenter prospective RVO study^15^, it was reported that there was no correlation between baseline SD-OCT parameters and BCVA changes. These results could be partially affected with inaccurate measurements by macular edema in the initial OCT images. As previously mentioned in SD-OCT image analysis^21^, thick CMT accompanying marked retinal edema or subretinal fluid (SRF) can make it challenging to determine the exact boundary of photoreceptor integrity. Chan et al.^17^ analyzed OCT images after 3 months of treatment and found that the initial 3-month evolution of DRIL was correlated with BCVA improvement. Similarly, the study by Mimouni et al.^16^ found that recovery of DRIL at 4 months was associated with better final BCVA improvement. In our study, we also found that the recovery of DRIL at the initial 3 months was a significant predictor of total BCVA improvement.

Our multivariate analyses showed that increased baseline ELM disruption, the presence of microaneurysms and p-MLM sign, and high serum total cholesterol level were risk factors of inhibiting a sustained extension of injection interval in BRVO-ME patients using the TAE regimen. Additionally, predictors of failure in maintaining the maximum interval at the last visit were diffuse baseline ELM disruption and the presence of microaneurysms.

Venous stasis in BRVO can lead to ischemic damage in the corresponding area, which increases the VEGF concentration and causes microaneurysm formation^22^. Microaneurysm formation has been identified as a risk factor for refractory BRVO-ME^23^. The p-MLM sign, an indicator of acute retinal ischemic damage, has also been associated with worse visual outcomes in CRVO^24^. In the current study, p-MLM signs along with microaneurysms were found to be risk factors for failures in maintaining a sustained extension of the treatment interval. This suggests that the E–F groups had relatively severe ischemic damage, and as a result, the VEGF concentration increased, making microaneurysms more likely to form. Further studies are needed to investigate the relationship between biomarkers indicating ischemic damage, such as VEGF levels of aqueous humor, and shortening of the treatment interval in BRVO-ME.

ELM functions as a barrier within the retina against large macromolecules, such as lipids and protein, from damaged retinal vasculature^25,26^. Therefore, much disruption of ELM will induce the refractory macular edema due to the migration of macromolecules within the inner retinal layers. Moon et al.^27^ have also reported that ELM disruptions in BRVO contribute significantly to refractory macular edema, which may require more frequent intravitreal injections. Earlier reports of diabetic macular edema^28,29^ have indicated that extravasation of lipids and proteins into the intercellular space of the retina most likely occurs as a result of disruption of BRB. In this study, it was observed that increased disruption of ELM and elevated serum total cholesterol levels were associated with a shorter treatment interval. This may be attributed to the possibility that elevated serum lipid levels can cause endothelium dysfunction^28^, leading to the exudation of macromolecules into the intercellular space via the disrupted barriers in the retina.

The current study had several limitations. First, the sample size was smaller than in other clinical trials. Second, a control group using the popular PRN regimen in the real world was not included. Third, imaging modalities such as OCT angiography, which can measure macular vessel density changes of retina, collateral vessels or microvascular abnormalities, were not evaluated in this study. Fourth, previous studies have shown that delayed treatment initiation is significantly associated with refractory ME^27,30^. However, since only acute onset of BRVO patients were enrolled in this study, we could not perform sub-analysis along the duration from diagnosis to treatment. Recent research has demonstrated the sustained benefits of a TAE regimen using aflibercept in patients with chronic CRVO, who had an average duration of 22 months. There was an improvement of 8 ETDRS letters in mean BCVA and a significant decrease in CMT compared to the baseline with reduced frequency of visits and treatments^31^. It is anticipated that similar outcomes can be achieved in cases of chronic BRVO. In addition, large-scale clinical studies of the TAE regimen compared to the PRN regimen involving parameters of OCT angiography are warranted.

Despite these limitations, our study has several strong points. First, to the best of our knowledge, this is the first prospective multicenter study to investigate risk factors associated with shortening of the injection interval in BRVO-ME under the TAE regimen. Second, by analyzing predictors for poor visual outcomes in BRVO-ME despite proactive aflibercept injections (to rule out the possibility of undertreatment such as PRN regimen), we identified useful predictors that could potentially achieve additional visual gain by switching to a more intensive strategy (e.i. monthly injection). Third, the multivariate analysis of predictors involved both SD-OCT parameters and laboratory findings, which could provide valuable insights for clinicians treating BRVO-ME patients using the TAE strategy.

In conclusion, in the treatment of BRVO-ME patients with the TAE regimen using aflibercept, the presence of microaneurysms and p-MLM sign, diffuse initial ELM disruption, and high serum total cholesterol levels may induce shortening of the treatment interval and a high treatment burden. In addition, better baseline BCVA, older age, and less recovery of the extent of DRIL for the initial 3 months indicated a low potential for visual improvement despite using the well-designed TAE regimen. Therefore, if a BRVO patient presents with the aforementioned risk factors, it could be reasonable to consider an early switch to a fixed monthly treatment regimen or to consider an initial monthly injection regimen for a longer period, as seen in other RCTs, to improve the visual prognosis.

## Methods

### Study design

This study was based on the PLATON trial^14^, which was a 72-week, multicenter, non-comparative, open-label clinical trial. This post-hoc analysis adhered to the tenets of the Declaration of Helsinki and was approved by the institutional review board of Yeungnam University Medical Center. All patients provided written informed consent. The treatment protocol has been described in detail previously^14^, and is briefly summarized here. Treatment consisted of intravitreal injections of 2 mg of aflibercept using a treat-and-extend protocol. All patients received three loading doses every 4 weeks. After the loading phase, the interval between study visits could be extended by 4 weeks at each visit, up to a maximum of 16 weeks, if there was a ≥ 10% decrease in CMT decrease compared to the previous visit. Treatment intervals were shortened by 4 weeks, to a minimum of 4 weeks, if there was a ≥ 10% increase in CMT compared to the previous visit and maintained at the current treatment if there was a < 10% change in CMT compared to the previous visit.

The E–F group included patients with ≥ 1 treatment interval decrease due to CMT increase compared to the previous visit after three monthly injections, including those who maintained their treatment intervals. In contrast, patients who continuously extended the treatment interval (maximum of up to 16 weeks) without extension failure were included in the E-S group. Additionally, patients were divided into two subgroups based on the injection interval maintained to 16 weeks or not at 52 weeks; the M-F group and the M-S group.

All patients underwent BCVA assessment measured by the ETDRS letter score, fundus examination, and SD-OCT (Spectralis; Heidelberg Engineering, Heidelberg, Germany) at each visit. We performed a post-hoc image assessment of SD-OCT images at baseline, three months, and 52 weeks. Clinical laboratory biomarkers such as lipid profile, and HbA1c were also evaluated at baseline.

### Participants

The study included men and women aged ≥ 18 years with center-involved ME secondary to BRVO for no longer than 3 months, who were treatment-naive and had documented BCVA based on an ETDRS letter score of 73–24 letters (Snellen equivalent of 20/40 to 20/320) in the study eye. Patients with diabetic retinopathy, uncontrolled diabetes mellitus (HbA1c > 7.0%), or hypertension (> 160 mmHg systolic or > 95 mmHg diastolic blood pressure) were excluded.

### Spectral-domain OCT image analysis

CMT was automatically measured by a software within the central 1-mm diameter of the ETDRS grid.^32^ The following parameters were assessed in the 1,500 μm wide area centered on the fovea: the amount of HF, horizontal size of the cyst, presence of epiretinal membrane and SRF. HF was defined as well-circumscribed, with approximately 20–40 μm dense particles with similar or higher reflectivity than the retinal pigment epithelium band on SD-OCT.^29,33^ The size of the cyst was measured based on the horizontal diameter of the largest cyst, similar to previous studies.^15,34^ The presence of microaneurysms and the p-MLM sign were evaluated in seven horizontal OCT B-scans within the central 1500 μm segment. The p-MLM sign was defined as a hyperreflective line located in the outer plexiform layer which is a reliable indicator of acute retinal ischemia on SD-OCT.^24^.

We assessed the DRIL and disruption of the ELM, EZ, and IZ within the central 1,500 μm segment using the method described by Sun et al.^35^ We analyzed the central seven horizontal OCT B-scans, which were spaced 120 μm between each B-scans:1 passing through the foveal center, 3 above, and 3 below it. The manual measurements using the caliper provided by an OCT software program in each of the seven B-scans were averaged. DRIL was defined as the disruption of any one of the two boundaries between the ganglion cell-inner plexiform layer, inner nuclear layer, and outer plexiform layer^17,35^. Two authors (W.S. and M.S.), who were blinded to all clinical information, interpreted all the above SD-OCT findings mentioned above. The average of the two measurements was considered.

### Statistical analyses

Statistical analyses were performed using IBM SPSS (version 22.0; IBM Corporation, Somers, NY, USA) and SAS (version 9.4; SAS Inc. Cary, NC, USA) software. The distribution of patient characteristics between the subgroups was compared using the Student’s *t* test. The correlation between BCVA and patient’s characteristics (biomarkers, SD-OCT parameters) including changes in SD-OCT parameters was analyzed by linear regression. Logistic regression analyses were used to assess the risk factors of frequent injection in the TAE regimen. *P* < 0.05 was considered statistically significant.

## Data Availability

The datasets generated during and/or analyzed during the current study are available from the corresponding author on reasonable request.
